# Towards a Transferable UAV-Based Framework for River Hydromorphological Characterization

**DOI:** 10.3390/s17102210

**Published:** 2017-09-26

**Authors:** Mónica Rivas Casado, Rocío Ballesteros González, José Fernando Ortega, Paul Leinster, Ros Wright

**Affiliations:** 1 School of Water, Energy and Environment, Cranfield University, Cranfield, Bedfordshire MK430AL, UK; paul.leinster@cranfield.ac.uk; 2 Regional Centre of Water Research, Universidad de Castilla-La Mancha, Carretera de las Peñas km 3.2, 02071 Albacete, Spain; rocio.ballesteros@uclm.es (R.B.G.); jose.ortega@uclm.es (J.F.O.); 3 National Fisheries Services, Environment Agency, Threshelfords Business Park, Inworth Road, Feering, Essex CO61UD, UK; ros.wright@environment-agency.gov.uk

**Keywords:** hydromorphology, intercalibration, unmanned aerial vehicle, photogrammetry, artificial neural network, water framework directive

## Abstract

The multiple protocols that have been developed to characterize river hydromorphology, partly in response to legislative drivers such as the European Union Water Framework Directive (EU WFD), make the comparison of results obtained in different countries challenging. Recent studies have analyzed the comparability of existing methods, with remote sensing based approaches being proposed as a potential means of harmonizing hydromorphological characterization protocols. However, the resolution achieved by remote sensing products may not be sufficient to assess some of the key hydromorphological features that are required to allow an accurate characterization. Methodologies based on high resolution aerial photography taken from Unmanned Aerial Vehicles (UAVs) have been proposed by several authors as potential approaches to overcome these limitations. Here, we explore the applicability of an existing UAV based framework for hydromorphological characterization to three different fluvial settings representing some of the distinct ecoregions defined by the WFD geographical intercalibration groups (GIGs). The framework is based on the automated recognition of hydromorphological features via tested and validated Artificial Neural Networks (ANNs). Results show that the framework is transferable to the Central-Baltic and Mediterranean GIGs with accuracies in feature identification above 70%. Accuracies of 50% are achieved when the framework is implemented in the Very Large Rivers GIG. The framework successfully identified vegetation, deep water, shallow water, riffles, side bars and shadows for the majority of the reaches. However, further algorithm development is required to ensure a wider range of features (e.g., chutes, structures and erosion) are accurately identified. This study also highlights the need to develop an objective and fit for purpose hydromorphological characterization framework to be adopted within all EU member states to facilitate comparison of results.

## 1. Introduction

A recognition of the importance of protecting and improving the water environment and the increasing pressures on water resources resulting from population growth, patterns of use and climate change has seen the introduction of major water protection laws in many countries. This includes the development of regulatory frameworks based on an assessment of the ecological quality of freshwater systems such as the European Union Water Framework Directive (EU WFD) [1], which aims to achieve good ecological status or potential of inland and coastal waters.

Each member state has adopted a specific methodology within the overall WFD to classify the ecological status of a water body based on an assessment of the biological, chemical, physico-chemical and supporting elements. The measures derived for individual parameters are based on significantly different methods and are therefore subject to multiple sources of difference. This variation in approach between member states means that it can be difficult to compare the results obtained in the various countries. In the particular case of supporting elements, recent studies have looked at the multiple methods used for hydromorphological characterization. Hydromorphology has a key role in the assessment of hydrology (i.e., the quantity and dynamics of water flow and connection to groundwater bodies), morphology (i.e., reach depth and width variation), structure and substrate of the river, structure of the riparian zone and river continuity [2].

The strengths and limitations in relation to the implementation of the WFD of a total of 139 available (European and non-European) hydromorphological characterization methods were identified under the REFORM project [3]. Overall, the majority of methods relied on the assessment of the physical habitat and did not provide sufficient consideration of physical processes or were pressure-specific. As a result, over the last few years there has been an increasing effort to develop geomorphologically based methods that facilitate the understanding of river functioning and evolution as a basis for assessing current conditions [4]. Examples of such methods are the Morphological Quality Index (MQI), the Morphological Quality Index for Monitoring (MQIm), the Geomorphic Units survey and classification System (GUS) [4,5,6,7,8,9] and the Hydromorphological Evaluation Tool (HYMET) [10].

The variables used for the characterization of hydromorphology depend upon the method being implemented. For example, the GUS system focuses on the identification of areas containing landforms created by erosion and or deposition inside or outside the river channel [6,9]. These can correspond to sedimentary units or include living or dead vegetation. Examples of geomorphic units are alternate bars, abandoned channels, cut-off channel, glides, island and riffles. The HYMET tool focuses on artificiality and sediment budget assessment [10]. The implementation of the method requires (i) the evaluation of the connectivity of the reach to the sediment production in its catchment; (ii) the analysis of the sediment transfer through the river network to the downstream reach and (iii) the quantification of the river sediment budget and artificiality. Variables such as the number of reservoirs present, the total reservoir storage volume and the quantity of produced sediment with free access to reach are estimated. More traditional methods such as the River Habitat Survey (RHS) [11] are based on descriptors of physical attributes (e.g., predominant bank material, predominant substrate within the channel, flow types and channel modification indicators).

Recent studies have looked at the harmonization of existing methods for hydromorphological characterization. Raven et al. [12] compared qualitatively three survey methods namely, the German Länder-Arbeitsgemeinschaft Wasser-Field Survey (LAWA-FS), the United Kingdom River Habitat Survey (RHS) and the French Système d’Evaluation de la Qualité du Milieu Physique (SEQ-MP). Differences in the quality assessment between the methods arose from the survey strategy and scale, data collection and analysis. Raven et al. [13] developed a methodology to test whether an UK hydromorphological assessment method (RHS) was suitable for rivers outside the UK. The aim was to assess whether the sampling scale (500 m) used was adequate and to suggest benchmarking strategies. Although some modifications were needed, the sampling scale was considered effective for the characterization of small rivers with the designation of hydro-ecological regions being identified as a key step towards a benchmarking program. Further work [14] compared the Ukrainian Field-Survey (UA-FS) and German LAWA-FS approaches to identify similarities and differences to the conformity of the outputs with the WFD requirements. Differences between methods included the assessment and interpretation of lateral erosion, sinuosity, type and depth profile, substrate diversity and special structures whereas similarities where observed for parameters describing land use, current diversity and water depth variation. Benjankar et al. [15] compared three hydromorphological characterization methods (i.e., the German LAWA, a special approach for urban rivers and a hydraulic based method), with results showing that assessment approaches developed for a certain geographic region may not be suitable for rivers in different contexts (i.e., ecoregion) and a transferable characterization approach was required. Langhans et al. [16] proposed a seven step procedure to combine assessment methods and harmonize metric outputs to a common scale from 0 to 1. The method was successfully tested for the integration of three hydromorphological methods developed in the USA (the Rapid Bioasessment Protocol-RBP), Switzerland (the Swiss Modular Concept of stream assessment-SMC) and Germany (LAWA) for four river sections. Similarly, in [2] a total of 121 hydromorphological assessment methods currently in use were compared to identify strengths, limitations and the potential to integrate different approaches. The results highlighted the need for an assessment approach based on integrated analysis, where the morphological and hydrological components drive the hydromorphological characterization. Fernandez et al. [17] reviewed more than 50 methods for river habitat characterization used worldwide. Results indicated that the key differences are the objectives for which the methods were designed, the time required for their implementation and whether the methods measure or evaluate characteristics. Overall, the parameters recorded most often in the various approaches were bank stability, channel substrate, artificial structures, riparian vegetation structure, channel dimensions, flow types, adjacent land uses and bars. The study concluded that methods that can be implemented at multiple spatial scales and provide quantitative information (i.e., indices vs. characterization protocols) were the most effective.

The WFD intercalibration exercise between member states [18] seeks to ensure comparability of ecological status boundaries and national assessment methods across Europe [19,20,21]. For this purpose, a set of six geographical intercalibration groups (GIGs) comprising waters of similar biogeophysical types (i.e., common intercalibration types) have been defined and are: Alpine, Eastern Continental, Central-Baltic, Mediterranean, Northern and Very Large Rivers (VLR) [18]. These are equivalent to the standard geographical stratification by ecoregion, where the ecoregions are geographical zones influenced by similar geophysical drivers.

The need to find pragmatic and cost-effective assessment approaches that can be adopted as common assessment methods across all countries or even as a baseline benchmark for the intercalibration exercise has been highlighted by some authors [19]. Recent studies have proposed the use of remote sensing data as a way to help achieve the harmonization of hydromorphological sampling protocols [2,22,23] at multiple spatial scales. Several tools have been developed to estimate key hydromorphological variables from remote sensing data. For example, [24] developed a method to derive valley width, active channel width and channel slope automatically from a 5 m spatial resolution DEM and 0.25–0.5 m spatial resolution orthoimage in a geographic information system (GIS) environment. Demarchi et al. [25] developed a semi-automated methodology for mapping hydromorphology indicators of rivers at regional scale using remote sensing data (i.e., high resolution near-infrared and LIDAR topography). The method enables the delineation of the natural fluvial corridor and primary riverscape units (e.g., water channel, unvegetated sediment bars, riparian densely-vegetated units) and in-stream mesohabitats. However, the resolution of commercially available remote sensing products (e.g., Lidar, aerial photography or synthetic aperture radar (SAR) data) is coarser than 10 cm and may not be sufficiently detailed for accurate hydromorphological characterization. Based on the work by [26], resolutions coarser than 2.5 cm provide biased characterizations of hydromorphological features when using artificial neural networks (ANNs) for their identification. The trade-off between high aerial imagery resolution and survey area coverage needs to be taken into account within this context. Aerial imagery at resolutions finer than 2 cm is difficult to obtain for wide-area characterization due to limitations in the current state-of-the art of the technology. Other limitations exist for commercially available remote sensing products: satellite data do not provide on-demand or temporally continuous data sets and provide oblique imagery which may result in partial spatial coverage when structures obstruct the view angle. In addition, both satellite and aircraft based remote sensing products fail to provide information under low cloud conditions.

Methodologies based on high resolution aerial imagery captured from Unmanned Aerial Vehicles (UAVs) have been proposed by several authors as potential approaches to overcome some of these limitations. For example, in [27] an UAV-based methodology for the automated recognition of hydromorphological features using ANNs was developed. Similarly, other authors [28,29] have identified photogrammetric methodologies based on high resolution UAV aerial imagery as the future tool of choice for reliable hydromorphological (i.e., physical river habitat) assessment. These techniques, albeit within their limitations [30], provide a rapid, inexpensive and increasingly accessible alternative to traditional remote sensing methods for physical habitat assessment whilst enabling quantitative microscale characterization [28]. The findings from these studies [26,27,28,30,31] raise the question as to whether frameworks based on the automated recognition of hydromorphological features from high resolution UAV aerial imagery could provide an objective and unbiased approach that can be adopted as a common assessment method across all EU member states and provide benchmark metrics for the intercalibration exercise.

In [27], we developed an UAV-based framework for the automated recognition of hydromorphological features and demonstrated an 81% average level of accuracy in feature classification for a 1.4 km reach (River Dee, Wales, UK). In [26], the level of resolution required for unbiased identification of hydromorphological features was determined for the same river reach and estimated to be 2.5 cm. Here, the aim is to assess the application of the framework to a range of different fluvial settings. This will be achieved through the following three core objectives:

Objective 1. To test the validity of the framework for a range of fluvial settings as identified by the WFD GIGs.
Objective 2. To compare the accuracy of the framework in hydromorphological feature identification within and between fluvial settings.
Objective 3. To interpret the outputs from (1) and (2) in line with the WFD regulatory framework.

## 2. Materials and Methods 

### 2.1. Study Sites

**Figure 1:**
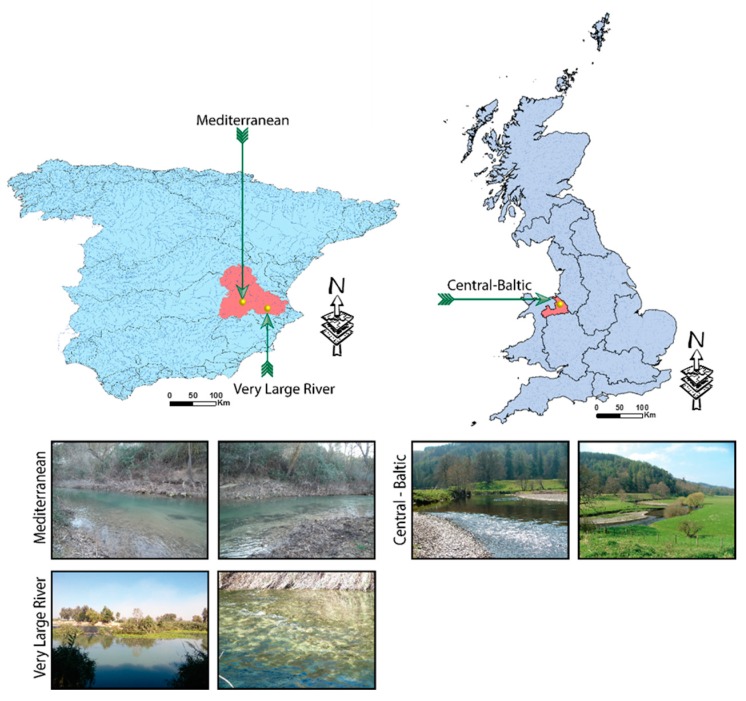
Schematic diagram showing the location of the selected study sites within each Geographical Intercalibration Group (GIG) and detailed imagery of the selected reaches. The maps of Spain and UK show the delineation of the main river basins with those basins containing the study sites highlighted in red.

Three sites corresponding to different GIGs (i.e., Central-Baltic, Mediterranean and VLR were selected for analysis (Figure 1 and Table 1). The sites were identified to maximize the diversity in hydromorphological characteristics along a ≈ 1 km reach and included the key hydromorphological features expected to be found in the named GIGs. From an initial set of reaches considered for analysis, only those that were representative of the spatial river variability within the waterbody where selected for further analysis. To ensure the selection was based on the current hydromorphological configuration of the waterbody, the full length of the river within each selected waterbody was assessed via multiple field-visits. The total reach length (≈1 km) was selected to exceed that used by the majority of the hydromorphological assessment methodologies used for WFD purposes, specifically those used in UK and Spain [2,3].

The Central-Baltic GIG was represented by a reach within the upper catchment of the River Dee, Wales, UK. The reach was located 30 km from the river source and 11.5 km downstream from the Bala reservoir. The river bed along the reach was characterized by gravel substrate with silt deposition in areas with low to non-perceptible flow. Bank erosion was visible along the reach with some sections presenting eroding cliffs. The banks were primarily covered by grazed grassland with few riparian trees scattered along the reach. The river length within the water body was 27.87 km [32], with the selected reach representing 5% of its totality. The overall designation for the reach was “heavily modified” [32].

The reaches representing both the Mediterranean and the VLR GIGs fell within the Jucar river basin in Spain. The Mediterranean GIG reach was located within the midland section of the river, near Motilleja (Albacete) and was characterized by a calcareous river bed with associated high entrenchment ratio. The reach contained small man-made structures that regulated the flow for irrigation purposes.

The majority of the calcareous bed channel had a layer of fine sediments due to intensive agricultural practices. The river length within the water body was 21.89 km [33,34], with the selected reach accounting for 5.5% of the total length. The overall WFD designation of the water body was “natural” [33,34].

The VLR GIG reach was located within the lower catchment of the Jucar, 500 m downstream of the Antella sluice (Antella, Valencia). The sluice diverted more than 25% of the river flow to irrigation [33,34]. Gabions were present along both banks for over 500 m at both the normal channel width and bankfull heights. The substrate within the reach was characterized by gravels with fine sediments intermittently present in some areas. The banks were dominated by invasive species (i.e., *Arundo donax*) with both submerged and emergent vegetation frequently present along the reach. The river length within the waterbody was 4.54 km [33,34], with the selected reach accounting for 26.4% of the total length. The WFD designation of the overall water body was “natural” [33,34].

### 2.2. Selection of Hydromorphological Variables

A set of variables that are major components in the WFD hydromorphological assessment in the UK and Spain were selected for automated identification (Table 2). These were specifically derived from: (i) the River Habitat Survey [11], the key method for the WFD hydromorphological assessment of rivers in UK; (ii) the UK methodology used for the designation of the WFD water body as heavily modified or artificial [36] and (iii) the equivalent Spanish methodologies [37,38,39,40,41]. The selection was based on the potential of these variables to contribute to the implementation of other WFD methods [2,3,5,7,8].

The scale at which the automated identification took place was an important consideration for the selection of hydromorphological features. This is because rivers are hierarchically organized systems; at each spatial and temporal scale there are a set of variables that are the most important in determining system behaviors and capacities [7,42]. The scale also determines the remote sensing data required for the characterization of hydromorphology [22]. Within this project, the variables selected focused on reach scale characterization which is the scale at which UAVs show optimal performance. The selected variables were divided into substrate, water, vegetation, shadows and artificial features.

Substrate features (i.e., side bars and erosion) provided information on the contemporary evidence of channel adjustment [7]. From a total of 121 hydromorphological characterization methodologies reviewed in [2], substrate related features were considered by 83 methods. Water features (i.e., riffles, glides, pools, shallow water, chute and major impacts) informed the characteristics and dimensions of the channel type and provide the physical base for habitats [7,9]. The identification of such features (i.e., flow type and change in depth [2]) was carried out within at least 34 of the hydromorphological characterization methods out of the 121 methods reviewed in [2]. Vegetation features (i.e., tree, vegetated side bar, vegetated bank, submerged free floating vegetation, emergent free floating vegetation, grass and nuisance plant species) provided evidence of the vegetation dynamics within the reach and allowed the estimation of derived parameters such as the percentage of riparian corridor under riparian vegetation or the riparian vegetation age structure [7]. They also provided information on the riparian vegetation continuity, species composition, coverage and distribution [2]. Belletti et al. [2] estimated that vegetation features (i.e., in channel vegetation and woody debris) were assessed within at least 54 hydromorphological characterization methods out of the 121 reviewed. Shadows were used to estimate the percentage of the channel with shade present whilst artificial features provided an indication of the constrains forced onto natural channel adjustments [7,9]. The presence of artificial features was recorded in over 80 methods out of the 121 reviewed in [2]. There was no clear indication of how many methods recorded shadows/shade was available.

### 2.3. Sampling Design and Data Collection

**Figure 2:**
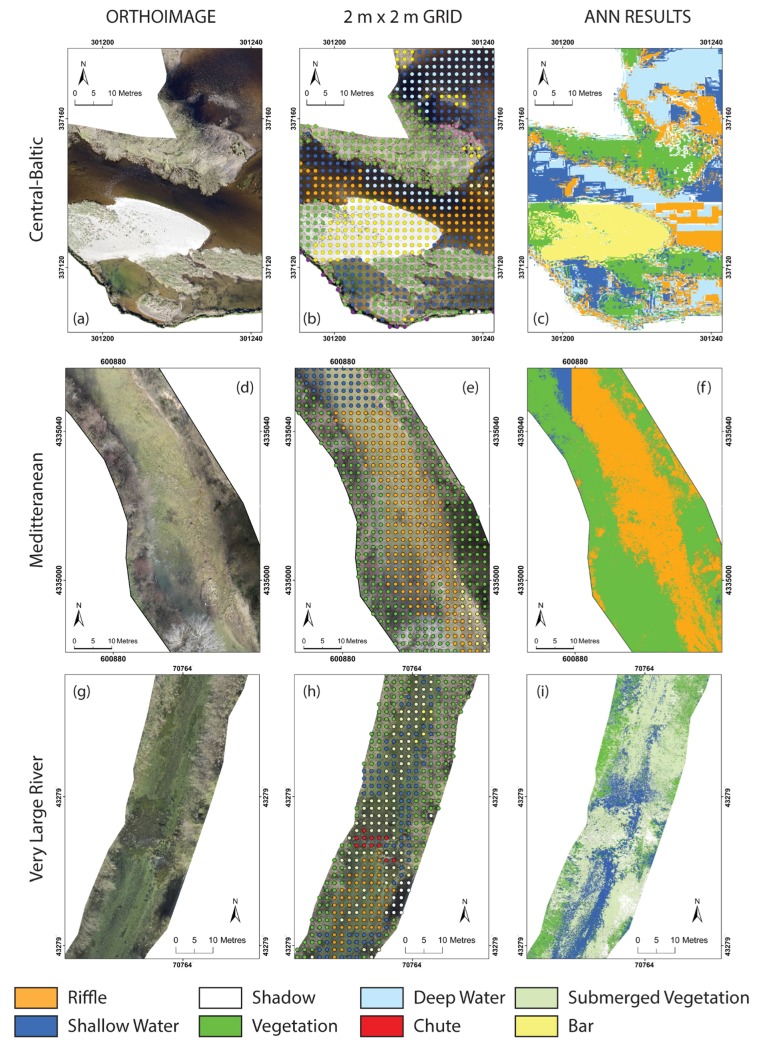
Example of classification outputs obtained for each Geographical Intercalibration Group (GIG). From left to right, orthoimage, 2 m × 2 m ground truth grid and classified outputs from the Artificial Neural Network (ANN); (**a**–**c**) Outputs for the Central-Baltic GIG reach; (**d**–**f**) Outputs for the Mediterranean GIG reach; (**g**–**i**) Outputs for the Very Large Rivers GIG reach.

**Figure 3:**
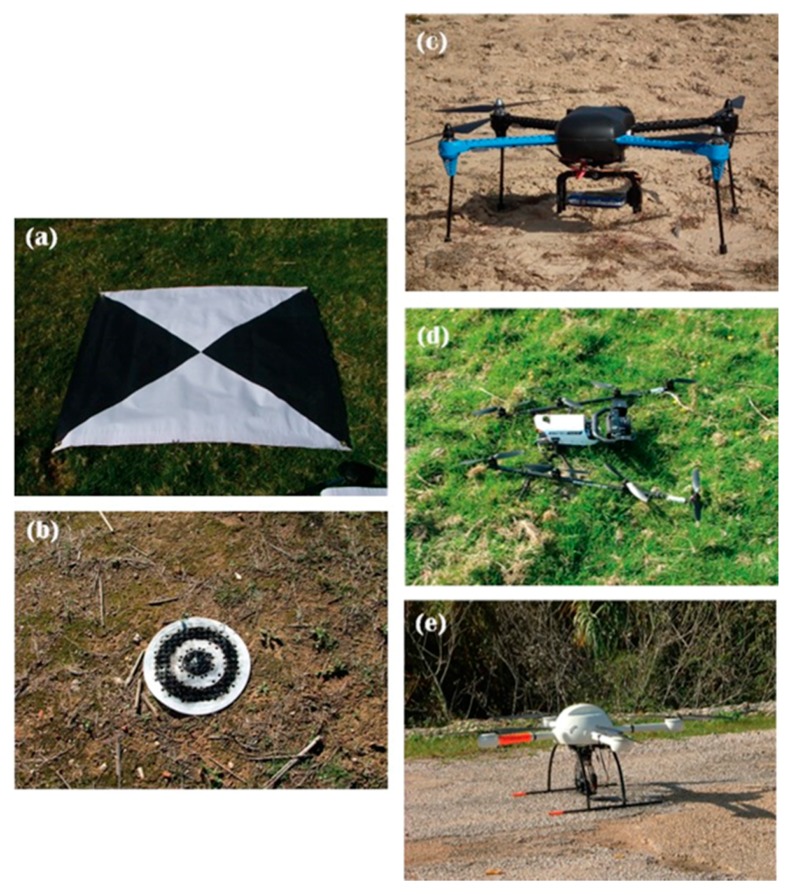
Types of Ground Control Points (GCPs) and Unmanned Aerial Vehicles (UAVs) used to collect the imagery at each reach. (**a**) 1 m × 1 m Squared GCP used in the Central-Baltic reach; (**b**) 0.30 m diameter GCP used in the Mediterranean and Very Large Rivers reaches; (**c**) IRIS9+ UAV (3DR, Berkeley, CA, USA) used at the Mediterranean reach; (**d**) Falcon 8 Trinity (ASCTEC, Krailling, Germany) used at the Central-Baltic reach; (**e**) md4-1000 UAV (Microdrones, Inc., Kreuztal, Germany) used at the Very Large Rivers reach.

Aerial imagery in the visible spectrum was collected over a 19-month period (21 Apirl 2015–24 November 2016) with a range of platforms and sensors specifically selected for the area to be surveyed (Table 3 and Figure 2 and Figure 3). Ground Control Points (GCPs) were deployed along the river banks to obtain parameters for external orientation. At the Central-Baltic GIG reach, the locations of the GCP centroids were obtained from a Leica GS14 Base and Rover Real Time Kinematic (RTK) GPS (Leica Geosystems AG, Heerbrugg, Switzerland) with a position accuracy of more than 0.02 m in the *X*, *Y* and *Z* dimensions. For all the other reaches, a Leica 1200 GPS linked to a GNSS permanent reference station with a position accuracy of 0.02 m in planimetry and 0.03 m in altimetry was used. Two different GCP designs were used in the study: 1 m × 1 m black squared GCPs with white opposite facing triangles where used for the Central-Baltic reach whereas white circular (0.30 m diameter) GCPs with concentric black rings were used for the Mediterranean and VLR reaches (Figure 2 and Figure 3).

Various vertical-take-off-and-landing UAV platforms (Figure 3) were used for image collection and included a Falcon 8 Trinity (ASCTEC, Krailling, Germany), an IRIS9+ (3DR, Berkeley, CA, USA) and a md4-1000 (Microdrones, Inc., Kreuztal, Germany). The Falcon 8 Trinity platform is a 0.77 m × 0.82 m × 0.12 m octocopter equipped with an uBlox LEA 6S GPS (uBlox, Thalwil, Switzerland). The platform has a vertical-take-off weight of 1.8 kg with the sensor payload (Sony Alpha) only requiring 0.34 kg. Flight endurance under optimal conditions and with fully charged lithium polymer (LiPo) batteries (6250 mAh) is 22 min. The IRIS 9+ is a 0.50 m diameter (from rotor hub to rotor hub) quadcopter equipped with a 3DR u-Blox GPS (uBlox, Thalwil, Switzerland) and a 0.4 kg payload capacity, with the camera requiring only 0.14 kg. Flight endurance under optimal conditions with the payload incorporated and with fully charged LiPo batteries (5100 mAh) is between 16–22 min. The md4-1000 is a 1.00 m diameter (from rotor hub to rotor hub) quadcopter with a 0.224 kg embedded camera, a total payload capacity of 1.2 kg and a maximum take of weight of 6 kg. The md4-1000 is equipped with an mdIMU and GNSS u-Blox6 positioning system. The flight endurance with fully charged 13,000 mAh LiPo batteries is 45 min. The ground sampling distance (GSD) for each reach was calculated to achieve a desired target imagery resolution of 2.5 cm based on the camera focal length and the regulatory airspace constraints within each country (Table 3). All flights were carried out by qualified UAV pilots following the country specific aviation regulation.

The methodology was first used and tested in the Central-Baltic GIG reach [26,27]. The lessons learnt from this deployment were transferred to the other reaches. Data were collected under low flow conditions and with a constant volumetric flow rate (Table 4). The number of flights required was determined by the platform, sensor and reach configuration. Each platform was configured to follow a flight plan that captured images at waypoints that achieved 60% along track and 40% across track image overlap. Longitudinal and cross-sectional multi-passes ensured full spatial coverage.

An ancillary data set was collected to aid visual identification of hydromorphological features. This consisted of a detailed rapid habitat map of the reach, associated photographic evidence of the records, accurate RTK GPS location of key features and measurements of water depth and velocity. The habitat map was obtained by walking along the reach and drawing polygons indicating the extent of the observed features described in Table 2. The RTK measurements and photographs were collected wherever key features were present to indicate their position and extent (subject to access). Both water depth and velocity readings were obtained by use of a radio controlled boat with integrated ADCP or a hand held ADCP and a standard surveying metric rod. All the information for a single reach was collected within a maximum of a five day period to ensure spatio-temporal collocation of multiple-sensor measurements.

### 2.4. Photogrammetry

**Figure 4:**
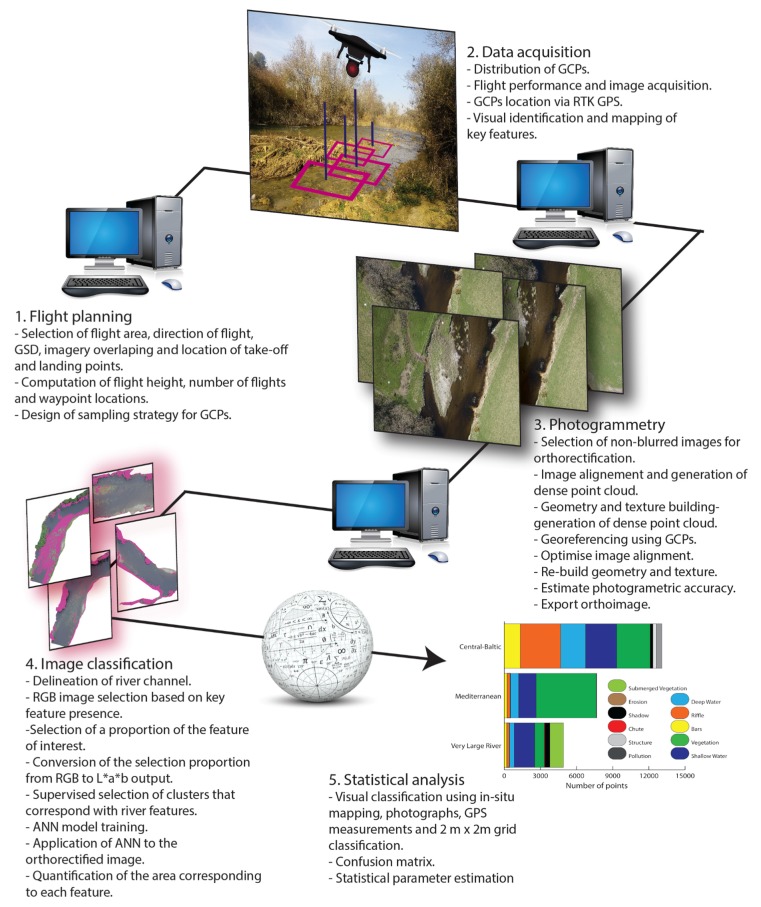
Workflow followed from imagery collection to multiple comparison analysis. ANN, GCP and GSD stand for artificial neural network, ground control point and ground sampling distance, respectively.

The imagery collected at each reach was assessed based on quality and spatial coverage for inclusion in the photogrammetric process (Figure 4, step 3). Blurred and distorted frames were excluded [46] for the generation of the standard geomatic products (i.e., orthoimage, digital terrain model and point cloud) via Photoscan Pro version 1.1.6 (Agisoft LLC, St. Petersburg, Russia). The photogrammetric process requires all the frames to be georeferenced (i.e., scale, translate and rotate) into a target Geodetic System (i.e., the World Geodetic System, WGS84) using the GCPs coordinates to minimize geometric distortion. For this purpose, the centroid of each GCP was manually identified in all frames and assigned the corresponding field RTK GPS coordinates. The processing time required for the photogrammetric process for each reach based on the performance of a computer with an Intel Core i7-5820k 3.30 GHz processor, 32 Gb RAM and two graphic cards (Geoforce GTX 980 and Qadro K2200, NVIDIA, Santaclara, CA, USA) is summarized in Table 3. The coregistration errors in *X*, *Y* and *Z* were automatically derived by Photoscan Agisoft the difference between the positions of the GCP centroids measured through RTK GPS and the coordinates derived from the imagery.

### 2.5. Automatic Classification

The automated identification of hydromorphological features was carried out via the Leaf Area Index Calculation (LAIC) software [47] (Figure 4, step 4), originally developed to discriminate green canopy cover from ground, stones and shadow background from high resolution aerial imagery [47,48]. LAIC bases the classification on an ANN approach (i.e., supervised classification technique) that segments the spectral domain of the RGB imagery into areas that directly relate to the features of interest. Key to the overall approach is the training process by which representative samples of target hydromorphological features are selected using a k-means clustering algorithm and used as the reference signature for the classification of the entire orthoimage.

The classification process was described in more detail in [27] but in brief, prior to the ANN training process, the RGB image was transformed into CIE *L* × *a* × *b* (CIELAB) space to perform a cluster analysis to identify the different river features. From the three parameters describing the CIELAB space (i.e., lightness (*L*), green to red scale (*a*) and blue to yellow scale (*b*)), only *a* and *b* were taken into account by the clustering algorithm. The number of clusters (*k*) depended on the feature being identified and was determined following an iterative process that increased *k* by one up to a maximum of ten clusters until visually satisfactory results were obtained. Within this context, visually satisfactory results required the image outputs to show that the feature of interest had been adequately identified. This supervised method was used as a basis to calibrate a Multilayer Perceptron (MP) ANN, which was applied to the remaining image. The ANN was based on three consecutive layers composed of inter-connected nodes. Within the context of this study, the outputs from the software were a classified map of the reach and estimates of the total area allocated to each of the hydromorphological features identified.

### 2.6. Statistical Analysis

The performance of the automated classification technique was assessed independently for each reach. The hydromorphological features were identified at each point of a 2 m × 2 m regular grid super imposed on each of the sampled reaches. The identification was based on the high resolution aerial imagery, the ancillary data sets encompassing the in-situ maps, the RTK measurements of key hydromorphological features and the photographic evidence of their distribution. The resulting output was considered to be the ground truth data set.

The performance of the ANN was assessed following [26,27] through confusion matrices and the estimation of derived standard metrics (Equations (1)–(5)) known as: overall accuracy (*AC*), true positive ratio (*TPR*), true negative ratio (*TNR*), false positive ratio (*FPR*) and false negative ratio (*FNR*). These were calculated as follows:

(1) $$AC=\overset{I}{\underset{i=1}{\sum }}\left(\frac{TN_{i}+TP_{i}}{TN_{i}+TP_{i}+FN_{i}+FP_{i}}\right)$$

(2) $$TPR_{i}=\frac{TP_{i}}{FN_{i}+TP_{i}}$$

(3) $$TNR_{i}=\frac{TN_{i}}{TN_{i}+FP_{i}}$$

(4) $$FNR_{i}=\frac{FN_{i}}{FN_{i}+TP_{i}}$$

(5) $$FPR_{i}=\frac{FP_{i}}{TN_{i}+FP_{i}}$$

where *TP_i_* (True Positives) is the number of points correctly identified as class *i*, *FN_i_* (False Negatives) is the number of points incorrectly rejected as class *i*, *TNi* (True Negatives) is the number of points correctly rejected as class *i*, *FP_i_* (False Positives) is the number of points incorrectly identified as class *i* and *I* is the total number of classes identified.

*TPR_i_*, *TNR_i_ FNR_i_* and *FPR_i_* were estimated for each of the features of interest whereas *AC* was a single value of overall classification performance. *AC*, as well as all the other ratios, ranged from 0 to 1. *TPR_i_* and *TNR_i_* quantifed the power of LAIC at classifying features correctly when compared to the ground truth whereas *FNR_i_* and *FPR_i_* showed misclassification rates.

## 3. Results

The image coregistration errors in X, *Y* and *Z* were below 2.7 cm for all reaches, with the VLR reach presenting the largest errors in *X* and *Y* (≈2.7 cm) and the smallest errors in *Z* (≈1 cm) (Table 5). The smallest coregistration errors in *X* and *Y* were found for the Mediterranean (≈1.06 cm) and the Central-Baltic (≈1 cm) reaches, respectively. The dimensions of the sampled reach (i.e., width and length) (Table 1) determined the number of points defined by the 2 m × 2 m regular grid and considered for analysis (Table 5). These varied between 4915 points (VLR) and 13,085 points (Central-Baltic).

**Figure 5:**
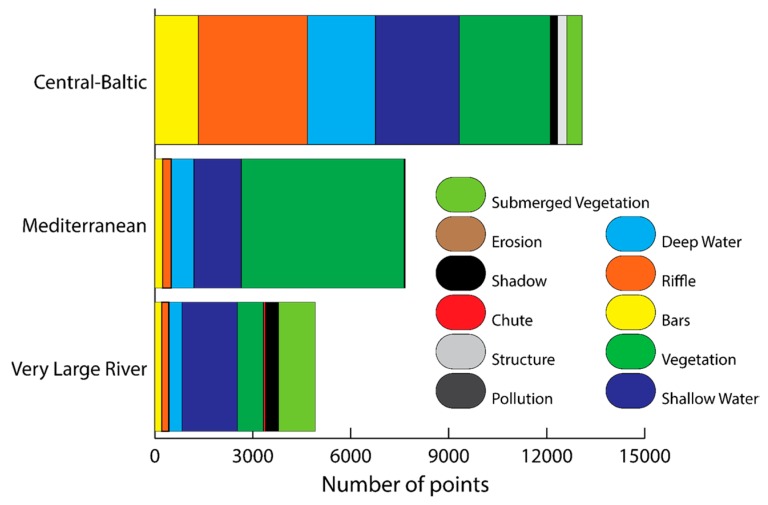
Number of points of the 2 m × 2 m ground truth grid allocated to each feature for each of the Geographical Intercalibration Groups (GIGs).

Based on the visual classification of the 2 m × 2 m grid, the type and extent of hydromorphological features varied considerably between reaches (Figure 5). The configuration within the Central-Baltic was predominantly dominated by riffles (26%), vegetation (25%), shallow waters (20%), deep water (16%) and bars (11%) with each of the other classes identified accounting for less than 3% of the total number of points. The dominant features within the Mediterranean reach include vegetation (65%), shallow water (19%) and deep water (10%) with all the other classes not accounting for more than 5% of the points each. These results were consistent with the pattern observed for the VLR reach, where vegetation, shallow water and deep water accounted for 39%, 35% and 8% of the points, respectively. Each of the remaining classes identified accounted for less than 8% of the classified points. The presence of shadows was significant along the Central-Baltic (≈2%) and VLR (8%) reaches with no shadows being identified along the Mediterranean reach.

The overall accuracy of the automated classification was above 50% for all the reaches, with the Central-Baltic reach presenting the best performance (81%), followed by the Mediterranean (71%) and the VLR (50%) reaches. The patterns of performance, as shown by the *TPR*, *TNR*, *FNR* and *FPR* values (Table 6), differed between reaches. Overall, the ANN was able to identify vegetation successfully with *TPR* values above 74% for all sites. Misclassification of vegetation features primarily occurred with riffles (Central-Baltic) and shallow waters (Central-Baltic, Mediterranean and VLR) (Table 6 and Table 7). Vegetation features were also classified as shadow (Central-Baltic and VLR) and deep waters (Mediterranean). These results were consistent with the performance of the ANN for the detection of riffles and side bars (*TPR* > 73% and *FNR* < 27% and *FPR* ≤ 6%) for the Central-Baltic and Mediterranean reaches. However, the performance in riffle and side bar identification decreased down to 0 for the VLR reach (*FNR* = 1). Overall, riffles were misclassified as shallow water (Central-Baltic and VLR), shadow (Central-Baltic), vegetation (Central-Baltic, Mediterranean and VLR) and deep water (VLR), whereas side bars were mainly misclassified as vegetation (Central-Baltic and VLR), riffles (Mediterranean) and shallow waters (Mediterranean and VLR) (Table 6 and Table 7).

For the specific case of deep waters, the ANN performance (*TPR*) was above 55% in all instances, with the best performances being observed in the Central-Baltic (*TPR* > 92%, *FNR* < 8%) and the VLR reaches (*TPR* > 66%, *FNR* < 34%). Misclassification occurred with riffles (Central-Baltic), shallow water (Central-Baltic and Mediterranean) and vegetation (Central-Baltic, Mediterranean and VLR) (Table 6 and Table 7). The performance in shallow water identification was consistent across the three reaches (*TPR* > 51%, *FNR* < 49% and *FPR* < 14%). Here, misclassification was present in shadows (Central-Baltic and VLR), vegetation (Central-Baltic, Mediterranean and VLR), riffles (Central-Baltic) and deep-water (Mediterranean) (Table 6 and Table 7). Shadows, when present, were successfully identified with *TPR*, *FNR* and *FPR* values >53%, <47% and <8%, respectively with misclassification mainly occurring with vegetation (VLR). Poor results were obtained across the three reaches when the ANN focused on the identification of chutes (*TPR* < 29%), erosion (*TPR* < 8%), structures (*TPR* = 0%) and major impacts (Table 2) such as pollution (*TPR* = 50%). The number of validation points available for these features was below 20 for the Mediterranean GIG site and 55 for the VLR GIG site (Table 7).

## 4. Discussion

The work reported in this paper aimed at assessing the transferability of an already tested framework for hydromorphological feature characterization [26,27] to a range of different fluvial settings. The three objectives addressed within this context were: (i) to test the validity of the framework to a range of fluvial settings as identified by the GIGs; (ii) to compare the accuracy of the framework in hydromorphological feature identification within and between fluvial settings and (iii) to interpret the outputs from (i) and (ii) in line with the WFD regulatory framework. The following sections discuss the outcomes within the context of these objectives.

### 4.1. UAV Framework Performance at the GIGs Sites

The proposed framework enables the identification of hydromorphological features with accuracies above 50% for all the GIGs considered. Overall, the ANN works robustly for the identification of vegetation, deep water, shallow water, side bars, riffles and shadows. For the particular case of the VLR GIGs, the ANN appears to underperform in the detection of both riffles and side bars. Riffles were systematically classified as deep water, shallow water or vegetation (submerged) whereas side bars were classified as vegetation or shallow waters. The underperformance detected for the VLR site may be the result of the configuration of the reach—i.e., transitional (from deep to shallow and vice versa) flowing water along a wide gravel bed channel that generated smooth rippled surfaces undetectable by the ANN. Within this setting, the ANN seem to be unable to detect the difference between riffles and shallow/deep waters. This is because all the riffles were present within the extent of the transitional water and could be classified as both feature categories simultaneously (e.g., riffles and shallow waters); any validation point falling under a riffle and being classified as shallow water (or vice versa) cannot be automatically considered a misclassification error.

The calcareous nature of the VLR reach may also affect the characteristics of the RGB aerial imagery captured; the water presented a low turbidity and a turquoise-like color that made feature identification (i.e., identification of differences in depth, velocity and substrate, amongst other parameters) challenging. This could explain why side bars (and riffles) were confused with vegetation as the color of both was similar under the survey conditions and why the distinction between riffles, deep and shallow waters cannot be accurately identified by the ANN. In [27], we explored in detail where the confusion between shallow and deep waters occurred along the Central-Baltic GIG site; the transition zone from deep to shallow areas were the key sources of feature misclassification. This lack of ability to identify the transition zones applies to all the reaches analyzed in the present study. The transition zone occupied larger proportions of the surveyed area in the Mediterranean and VLR reaches.

The ANN performs poorly on the classification of chutes, artificial structures, erosion and pollution. These features appeared occasionally along some of the reaches and were not representative enough for the ANN to register them as independent clusters (i.e., they accounted for less than 8% of the classified points). In the particular case of pollution, the ANN provides encouraging results (TPR > 53%). However, the limited number of validation points (i.e., 16 points along the Mediterranean reach) available for that particular feature class does not allow for a conclusive statement on the performance of the ANN for pollution identification. Note that pollution appeared in the Mediterranean GIG reach only and along a backwater that collected white foam and plastics derived from upstream activity.

### 4.2. Potential Technical Improvements 

For the proposed framework to be used for large scale (>1 km) hydromorphological characterization, it is necessary to increase its cost-effectiveness via the reduction of the number of GCP to be deployed. Recent work by several authors (e.g., [48,49]) has already contributed to address this gap in knowledge. This, coupled with increased UAV battery performance, data capture and less CPU demanding software makes UAV based frameworks a plausible option for wide-area (>1 km reach) robust and accurate hydromorphological assessment. Further work should compare the performance of the proposed framework with existing classification techniques for geomorphological environments (e.g., [50,51]).

The work presented here focuses on the characterization of hydromorphological variables at reach level. It is not yet certain whether the framework can be applied to larger spatial scales (e.g., catchment [7]) and achieve the same efficiency as current remote sensing methodologies [22]. Woodget et al. [30] identify the spatial coverage as a key challenge for the routine operational use of UAVs and digital photogrammetry for river habitat mapping. The strong trade-off between resolution and spatial coverage [30] suggests that some aspects of the hydromorphological characterization for WFD purposes may be achieved from UAV imagery but that complementary remote sensing methods may be required to address the remaining aspects [22]. For example, the strong reliance on image texture required for the implementation of Structure-from-Motion (SfM) may also compromise the wide-area implementation of the framework. If the texture of the imagery is compromised, SfM will not detect matching features between overlapping images and fail to produce an orthoimage of the surveyed area [30]. Further research is therefore required to address these points before the framework can be adopted for wide-area monitoring. In addition, further consideration needs to be given to the type of sensors to use on the UAV platform. The RGB imagery collected for this study enables the qualitative assessment of hydromorphological features (e.g., deep/shallow waters) but does not facilitate the quantitative estimation of depth and velocity within the channel reach [30]. It has been highlighted by some authors that the RGB imagery presents limited radiometric resolution which obstructs the restitution of topography in darker parts (e.g., shadow and deep water) [30]. Sensors able to provide multispectral and RAW format imagery may be beneficial and should be considered in further developments of the framework. The variability in the quality of the geomatic products generated from high resolution UAV aerial imagery has also been raised as a general concern by some authors [52]. For example, the optimal selection of GCP can improve the variability of the Digital Elevation Model from 37 mm to 16 mm. The magnitude of these changes in the DEM may be relevant when changes in hydromorphological characteristics over time are required [52] but not relevant where regulatory assessments are the primary consideration.

### 4.3. Framework Output Interpretation in Line with the WFD

The results obtained indicate that the methodology can be successfully transferred within and between river GIG types and countries but that water turbidity, color and underlying river bed substrate may affect classification performance. The hydromorphological features successfully identified (i.e., vegetation, deep water, shallow water, side bars, riffles and shadows) are common to the majority of hydromorphological characterization methods reviewed in [2] and are therefore of great relevance for the transferability of this framework. They will be able to contribute to the implementation of the WFD in UK as they align with the descriptors included in the RHS methodology [11] and the designation of WFD artificial and heavily modified water bodies [36]. Similarly, they will aid the implementation of the Spanish hydromorphological assessments [37,38,39,40,41] and contribute to the implementation of WFD methodologies in other countries [2,3,5,7,8].

The framework outlined in this paper is a step forward towards the accurate identification of in-channel and bank features and could be integrated with complementary frameworks. Recent work by other authors have shown that other riverine features can be identified with similar UAV based methodologies. In [53], supervised machine learning approaches were used to identify different types of macrophytes. In [54], object oriented classification was used to map dead wood presence, whereas in [29], geomatic products derived from UAV aerial imagery were used to characterize channel substrate. Further research should focus on integrating these scientific advances into a single methodological framework that enables the objective and automated characterization of key WFD water elements. This will address the need identified in previous works [2] for the development of an integrated hydromorphological analysis that includes both morphological and hydrological components to evaluate and classify hydromorphological state and quality. In turn, this will contribute to addressing some of the current limitations in the WFD intercalibration exercise.

At present it is not possible to identify fully the consequences of the EU member states using different methodological approaches to assess the ecological status of water bodies. The intercalibration exercises have not provided a clear answer yet [14,21] and as a result, there is still insufficient information about how the different methods should be implemented and integrated to achieve readily comparable assessments [20]. It is possible that the consequences will be both financial and legal in terms of the measures and efforts undertaken to achieve good ecological status or potential under the WFD [12,14]. An objective and fit for purpose hydromorphological characterization framework that is widely adopted within all member states will facilitate comparison of the results obtained across Europe and will provide a level playing field. The UAV based framework presented here would contribute for example, to a more consistent designation and overall hydromorphological assessment by addressing some of the current limitations, namely: inadequate characterization of pressure gradients within the GIGs, difficulty comparing results obtained from methods with different metrics, lack of comparable data sets and implementation of subjective methods [14] through the provision of a standardized approach to understanding the hydromorphological quality in rivers.

An interesting application of the framework described in this paper would be its use for the designation of the waterbody as “natural”, “heavily modified” or “artificial”. Currently such designations can be influenced to a significant extent by management and external influence. For the case study areas, the rivers studied representing the Mediterranean and VLR GIGs had been designated by the Spanish authorities as having “natural” hydromorphological conditions [33]. In the UK, the reach representing the Central-Baltic GIGs was less modified than the Spanish reaches but had been designated by the authorities as “highly modified” [35]. The differences in the approaches taken for the designation of the waterbodies translates into different requirements in terms of the measures to be implemented and the associated level of investment required to improve the ecological status of the water bodies. This mismatch in the implementation of the WFD could be addressed if a common framework for hydromorphological characterization were to be adopted. The techniques developed could also be useful in a number of situations where hydromorphological characterization is required aside from the WFD implementations such as river restoration appraisal [55].

## 5. Conclusions

This paper reports the transferability of an already validated ANN based framework for hydromorphological characterization of three different fluvial settings as defined by the WFD GIGs. The framework provides overall accuracies greater than 50% for all the reaches but shows some limitations when applied to calcareous rivers falling under the VLR GIG. Deep waters, shallow waters, vegetation, side bars, riffles and shadows are successfully identified within all reaches, with the ANN underperforming on the identification of side bars and riffles for the VLR GIG. Further work is required to develop more reliable algorithms that can be incorporated into the existing ANN for the accurate detection of a wider range of features, including chutes, erosion, structures and major impacts (i.e., pollution). This work also highlights the need to develop objective and reliable hydromorphological assessment frameworks that are widely adopted by all EU member states. The framework presented here is a step forward towards that goal.

## Figures and Tables

**Table 1:** Characteristics of the selected reaches within each of the Water Framework Directive Geographical Intercalibration Groups (GIGs). WFD D, WB ID, WB L, RL, RW and HMWB stand for Water Framework Directive Designation, Water Body Identification code, Water Body Length, Reach Length, mean wetted Reach Width and Heavily Modified Water Body, respectively. VLR stands for Very Large Rivers GIG. Area (m^2^) refers to the total area within which hydromorphological features were identified. RW was estimated based on a total of 20 width measurements randomly taken along the reach. ^1^ [35] and ^2^ [33].

	Geographical Intercalibration Group		
Descriptor	Central-Baltic	Mediterranean	VLR
River	Dee	Jucar	Jucar
Country	UK	Spain	Spain
WFD D	HMWB ^1^	Natural ^2^	Natural ^2^
WB ID	GB111067052240 ^1^	ES080MSPF18.12 ^2^	ES080MSPF18.28 ^2^
WB L (km)	27.73 ^1^	21.89 ^2^	4.54 ^2^
RL (km)	1.4	1.2	0.96
RW (m)	32.62	11.09	18.78
Area (m^2^)	46,385	30,859	21,784

**Table 2:** Description for each of the hydromorphological features identified within the selected study sties. The features are adapted from the River Habitat Survey [11], the key method for the hydromorphological assessment of rivers in UK and for the designation of the water body [36]. This was further supported by the features used by the Spanish methodologies [37,38,39,40,41]. The water features recorded extended for over 5 m or >1% of the channel length following [11]. These features were recorded even if they were the result of an artificial structure.

Feature		Description
Substrate	Side Bar	Consolidated river bed material along the margins of a reach which is exposed at low flow.
	Erosion	Predominantly derived from eroding cliffs which are vertical or undercut banks, with a minimum height of 0.5 m and less than 50% vegetation cover.
Water	Riffle	Area within the river channel presenting shallow and fast-flowing water. Generally over gravel, pebble or cobble substrate with disturbed (rippled) water surface.
	Deep Water (Glides and Pools)	Deep glides: deep homogeneous areas with visible flow movement along the surface. Pools: localized deeper parts of the channel created by scouring. Both features present fine substrate, non-turbulent and slow flow.
	Shallow Water	Includes any slow flowing and non-turbulent areas.
	Chute	Low curving fall in contact with substrate.
	Major impacts (pollution)	Indicators of water quality pollution (e.g., accumulation of white/sluggish foam, tipping, litter, sewage, abstraction).
Vegetation	Tree	Trees obscuring the aerial view of the river channel. The distinction between perennial and tree in dormant period was made when possible.
	Vegetated Side Bar	Side bar presenting plant cover in more than 50% of its surface.
	Vegetated Bank	Banks not affected by erosion. When possible the difference was made between grass and shrub cover.
	Submerged Free Floating Vegetation	Plants rooted on the river bed with floating leaves.
	Emergent Free Floating Vegetation	Plants rooted on the river bed with floating leaves on the water surface.
	Grass	Present along the banks and floodplain as a result of intense grazing regime.
	Nuisance plant specie	Invasive species covering a large proportion of the banks or river channel.
Shadows		Extent of direct, overhead, tree canopy shade. Includes shading of channel and overhanging vegetation.
Artificial		Any weir, sluices, culverts, bridges, fords, deflectors or equivalent that are not underwater.

**Table 3:** Key characteristics of the platforms and sensors used to gather the imagery at each river reach. GIG, VLR, GCP, GSD, Mill. effect. pix. and FLA stand for Geographical Intercalibration Group, Very Large Rivers, Ground Control Point, Ground Sampling Distance, Million Effective Pixels and Focal Length Applied, respectively. The time required for the photogrammetric process (PT) is estimated based on the performance of a computer with an Intel Core i7-5820k 3.30 GHz processor, 32 Gb RAM and 2 graphic cards (Geoforce GTX 980 and Qadro K2200, NVIDIA, Santa Clara, CA, USA). ^1^ Sony Corporation, Tokio, Japan. ^2^ Canon^TM^, Tokio, Japan.

GIG	Central-Baltic	Mediterranean	VLR
GCPs	60	20	8
GSD	2.5	2.17	2.21
Flight altitude	100	77.6	120
No. Flights	4	2	2
Platform	Falcon 8 Trinity	IRIS9+	md4-1000
Camera	Sony Alpha 6000 ^1^	Canon IXUS 115 HS ^2^	Sony Alpha ILCE-5100 ^1^
Sensor type	CMOS APS-C type Exmor^TM^ HD ^1^	BCI-CMOS ^2^	CMOS APS-C type Exmor^TM 1^
Mill. effect. pix.	24.3	12.1	24.3
Pixel size (mm)	0.00391	0.02169	0.02214
FLA (mm)	20	5	20
PT (h)	12	12	12

**Table 4:** Description of weather and reach characteristics during the flight at each of the Geographical Intercalibration Groups (GIGs) study sites. Weather conditions at the Central-Baltic reach were estimated based on the Shawbury (Shropshire, UK) meteorological aerodrome report. Similar information was obtained from the Agroclimatic Information for Irrigation Service weather stations at Motilleja (Albacete, Spain) and Xátiva (Valencia, Spain) for the Mediterranean and Very Large Rivers (VLR) GIGs reaches, respectively. Q stands for flow. ^1^ [43], ^2^ [44], ^3^ [45].

GIG	Central-Baltic ^1^	Mediterranean ^2,3^	VLR ^2,3^
Date	21 Apirl 2015	28 January 2016	24 November 2016
Discharge (m^3^ s^−1^)	4.8	2.5	3.4
Percentile Q (m^3^ s^−1^)	Q80	Q80	Q80
Surface wind	1 m s^−1^–3 m s^−1^	0.46 m s^−1^	0.93 m s^−1^
Wind direction	60–350°	307°	293.5°

**Table 5:** Parameters describing the coregistration errors and the overall performance in hydromorphological feature identification for each of the Geographical Intercalibration Group (GIG) sites. N and AC stand for the number of points in the 2 m × 2 m grid and the accuracy in feature classification. GCP and VLR stand for Ground Control Point and Very Large Rivers, respectively.

GIG	Central-Baltic	Mediterranean	VLR
Total GCP error in *X* (cm)	1.1	1.06	2.65
Total GCP error in *Y* (cm)	1.0	1.49	2.52
Total GCP error in *Z* (cm)	1.6	1.42	1.01
*N*	13,085	7716	4915
*AC* (%)	81	71	50

**Table 6:** Summary of the ANN performance in hydromorphological feature identification per Geographical Intercalibration Group (GIG) and feature. *TPR*, *TNR*, *FPR* and *FNR* stand for true positive ratio, true negative ratio, false positive ratio and false negative ratio, respectively.

Feature	*TPR*	*TNR*	*FPR*	*FNR*
**Central-Baltic**				
Side bar	0.822	0.765	0.000	0.178
Erosion	0.077	0.786	0.001	0.923
Riffle	0.814	0.756	0.060	0.074
Deep water	0.926	0.741	0.008	0.074
Shallow water	0.588	0.815	0.051	0.412
Shadow	0.818	0.770	0.073	0.182
Vegetation	0.810	0.758	0.081	0.192
**Mediterranean**				
Side bar	0.758	0.706	0.000	0.241
Riffle	0.736	0.707	0.014	0.263
Deep water	0.550	0.724	0.044	0.449
Shallow water	0.515	0.753	0.093	0.484
Vegetation	0.785	0.565	0.299	0.214
Pollution	0.500	0.708	0.001	0.500
Structure	0.000	0.709	0.000	1.000
Chute	0.000	0.708	0.000	1.000
**Very Large Rivers**				
Side bar	0.000	0.524	0.002	1.000
Riffle	0.000	0.527	0.000	1.000
Deep water	0.665	0.488	0.034	0.334
Shallow water	0.555	0.475	0.136	0.444
Shadow	0.531	0.500	0.073	0.468
Vegetation	0.743	0.481	0.364	0.256
Structure	0.000	0.503	0.000	1.000
Chute	0.283	0.505	0.003	0.716

**Table 7:** Confusion matrix obtained per Geographical Intercalibration Group (GIG) site and feature considered. The features identified are described in Table 4 and are as follows: Side Bars (SB), Riffles (RI), Erosion (ER), Deep Water (DW), Shallow Water (SW), Chute (CH), Shadow (SH), Vegetation (VG), Pollution (PL), Structure (ST) and Georeferencing Error (GE). ANN refers to the features identified with the Artificial Neural Network algorithms. Visual refers to the features identified through visual observation and considered to be the ground truth data set.

	ANN	SB	RI	ER	DW	SW	CH	SH	VG	PL	ST	GE	Total
Visual													
**Central-Baltic**													
**SB**		1097	8	-	-	2	-	10	214	-	-	3	1334
**RI**		-	2717	1	-	318	-	219	76	-	-	8	3339
**ER**		-	13	22	1	3	-	10	238	-	-	-	287
**DW**		-	60	-	1927	54	-	8	29	-	-	4	2082
**SW**		-	262	-	80	1514	-	493	217	-	-	7	2573
**CH**		-	-	-	-	-	-	-	-	-	-	-	-
**SH**		-	-	4	-	5	-	180	31	-	-	-	220
**VG**		-	245	11	7	156	-	197	2129	-	-	9	3250
**PL**		-	-	-	-	-	-	-	-	-	-	-	-
**ST**		-	-	-	-	-	-	-	-	-	-	-	-
**Total**		1097	3305	38	2015	2052	-	1117	3430	-	-	31	13,085
**Meditteranean**													
**SB**		176	19	-	-	16	-	-	-	-	-	21	232
**RI**		-	198	-	-	1	-	-	65	-	-	5	269
**ER**		-	-	-	-	-	-	-	-	-	-	-	-
**DW**		-	-	-	385	77	-	-	188	-	-	47	697
**SW**		-	25	-	59	752	-	-	541	-	-	71	1448
**CH**		-	-	-	-	2	-	-	1	-	-	-	3
**SH**		-	-	-	-	-	-	-	-	-	-	-	-
**VG**		-	61	-	248	495	-	-	3919	14	-	253	4990
**PL**		-	-	-	-	-	-	-	5	8	-	3	16
**ST**		-	-	-	-	-	-	-	1	1	-	8	10
**Total**		176	303	0	692	1343	-	-	4720	23	-	408	7665
**Very Large River**													
**SB**		-	-	-	-	49	-	5	150	-	-	22	226
**RI**		-	-	-	104	75	1	1	44	-	-	5	230
**ER**		-	-	-	-	-	-	-	-	-	-	-	-
**DW**		-	-	-	268	4	-	12	119	-	-	25	428
**SW**		7	-	-	3	942	9	137	597	-	-	116	1811
**CH**		-	-	-	12	-	15	4	22	-	-	50	103
**SH**		-	-	-	1	36	-	214	152	-	-	402	805
**VG**		6	-	-	35	276	5	171	1431	-	-	551	2475
**PL**		-	-	-	-	-	-	-	-	-	-	-	-
**ST**		-	-	-	-	-	-	3	5	-	-	1	9
**Total**		13	-	-	423	1382	30	547	2520	-	-	1172	6087
